# Concurrent isolated retroperitoneal HGSC and STIC defined by somatic mutation analysis: a case report

**DOI:** 10.1186/s13000-019-0795-3

**Published:** 2019-02-11

**Authors:** Kazuaki Suda, Hirofumi Nakaoka, Chihiro Hata, Natsumi Yahata, Masanori Isobe, Hitoshi Kameyama, Toshifumi Wakai, Teiichi Motoyama, Ituro Inoue, Kosuke Yoshihara, Takayuki Enomoto

**Affiliations:** 1 Department of Obstetrics and Gynecology, Niigata University Graduate School of Medical and Dental Sciences, 1-757 Asahimachi-dori, Niigata, 951-8510 Japan; 2 Department of Genetics, School of Life Sciences, Graduate University for Advanced Studies (SOKENDAI), Hayama, Japan; 3 Division of Human Genetics, National Institute of Genetics, Mishima, Japan; 4 Department of Obstetrics and Gynecology, Niigata Prefectural Shibata Hospital, Shibata, Japan; 5 Division of Digestive and General Surgery, Niigata University Graduate School of Medical and Dental Sciences, Niigata, Japan; 6 Department of Molecular and Diagnostic Pathology, Niigata University Graduate School of Medical and Dental Sciences, Niigata, Japan

**Keywords:** Retroperitoneal high-grade serous carcinoma, Serous tubal intraepithelial carcinoma, Somatic mutation, Case report

## Abstract

**Background:**

Retroperitoneal high-grade serous carcinoma (HGSC) is extremely rare and the origin remains unclear. We present a case of retroperitoneal HGSC and coexisting serous tubal intraepithelial carcinoma (STIC), which is considered as the main origin of ovarian HGSC. We reviewed the available literature and discussed about the origin of this rare disease.

**Case presentation:**

A 58-year-old female with a 93 × 65 × 62 mm-solid tumor with a cystic part was located immediately dorsal to the rectum underwent bilateral salpingo-oophorectomy, total abdominal hysterectomy, and en bloc resection of the retroperitoneal tumor together with lower anterior resection of the rectum. Histological diagnosis was retroperitoneal HGSC and STIC at the right fallopian tube. Two deleterious somatic mutations in *TP53* and *BRCA2* genes were shared between retroperitoneal HGSC and STIC.

**Conclusions:**

In addition to clinical features in the previous reports, our genetic findings suggest the origin of retroperitoneal HGSC might be STIC.

## Background

High-grade serous carcinoma (HGSC) is the most common histological type of ovarian cancer. Since genetic relationships between intraepithelial carcinoma of the fimbria and pelvic HGSC were clarified in 2007 [1, 2], a paradigm shift in the origin of ovarian cancer has been occurring. At present, serous tubal intraepithelial carcinoma (STIC) is recognized as the precursor lesion of HGSC [3–5], and a recent genomic study also endorses the theory that HGSC originates in fallopian tube [6]. In fact, the knowledge that STIC cells migrate onto the peritoneum corresponds to the clinical feature of HGSC characterized by peritoneal dissemination and massive ascites.

On the other hand, retroperitoneal HGSC is very rare case [7–13] and the precursor of retroperitoneal HGSC remains unclear. In this case report, we present a case of HGSC entirely existing in the retroperitoneal space without the evidence of lymphovascular invasion. Furthermore, we demonstrate that retroperitoneal HGSC and coexisting STIC shared the same somatic mutations using next-generation sequencing.

## Case presentation

**Fig. 1:**
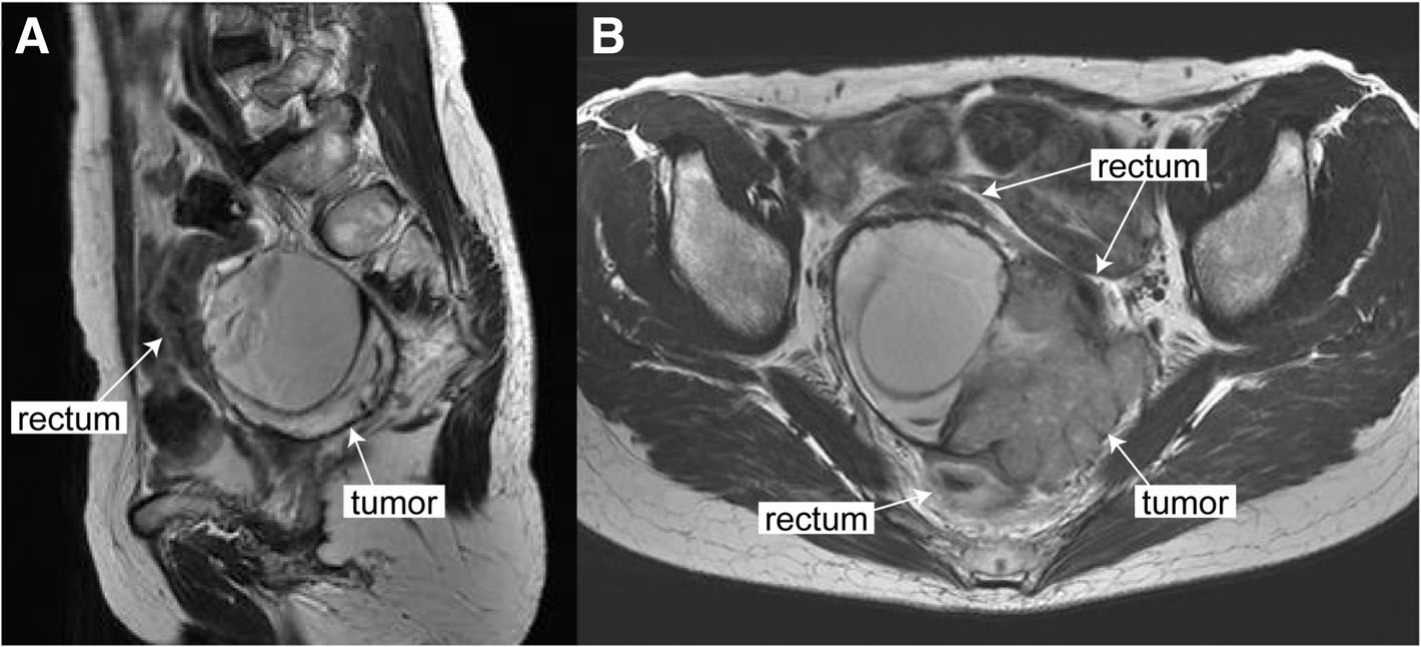
Preoperative T2-weighted magnetic resonance imaging showed the pelvic mass composed of cystic and solid parts in **a** axial and **b** sagittal views. Arrowheads indicate the tumor and rectum in each panel

**Fig. 2:**
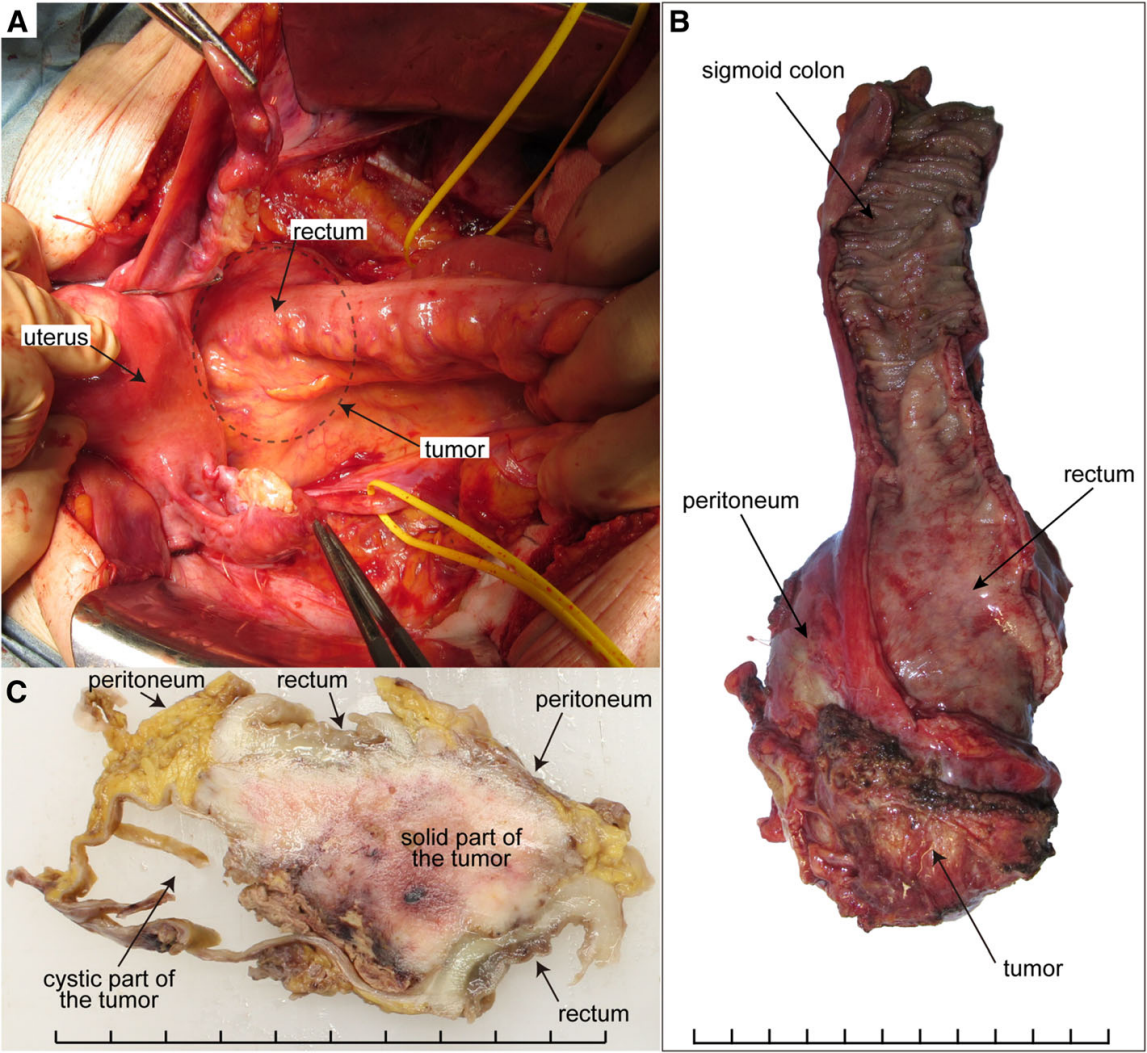
**a** Pelvic laparotomy revealed the rectum compressed by the tumor in the mesorectum. The tumor was lying beneath the rectum at the area of dotted circle. Bilateral ureters were marked with yellow tapes. **b** The retroperitoneal capsuled tumor was resected with the rectum and the part of sigmoid colon. The tumor was surrounded by the rectum as a wrap. **c** A cross-sectional photograph of the surgical specimen. The tumor was completely separated from the peritoneal cavity with the rectum and peritoneum. Divisions of the scale bar in each panel show one cm

A 58-year-old (gravida 2, para 2) woman presented the nearby hospital complaining of persistent defecation disorder and vomiting. Although her family history was notable for pancreatic cancer in her father, there was no other familial history of cancer, including breast and ovarian cancer. Her past medical history is unremarkable. Her past surgical history includes right ovarian cystectomy for a dermoid cyst at the age of 30. A computed tomography (CT) scan showed a large pelvic tumor adjacent to the rectum. Laboratory findings showed that her serum level of cancer antigen (CA) 125 increased to 315.2 IU/ml. Magnetic resonance imaging (MRI) demonstrated that a 93 × 65 × 62 mm-solid tumor with cystic parts was located immediately dorsal to the rectum (Fig. 1). CT and MRI showed no evidence of dissemination, lymph node metastasis, nor distant metastasis. Colonoscopy showed strong extrinsic compression at the rectum with intact mucosa; however, biopsy of the rectum and the tumor site was not performed during colonoscopy. Based on the MRI finding that a perirectal cystic tumor was present without peritoneal dissemination, stage IA ovarian cancer was suspected, and she was referred to our hospital for treatment. At laparotomy, the tumor was located dorsal to the rectum and existed entirely in the retroperitoneal space (Fig. 2a). There were no apparent lesions in the peritoneal cavity including bilateral adnexa, uterus, and peritoneum. Peritoneal washing cytology was negative. After bilateral salpingo-oophorectomy and total abdominal hysterectomy, en bloc resection of the retroperitoneal tumor together with lower anterior resection of the rectum was performed (Fig. 2b). Whereas the tumor was adhered to the rectal wall, the tumor itself was relatively well-capsulated and easily separated from surrounding fat tissues. Based on pathological diagnosis of the retroperitoneal tumor: high-grade serous carcinoma, she received 6 cycles of adjuvant chemotherapy with carboplatin, paclitaxel and bevacizumab according to the standard treatment strategy for ovarian cancer. After the combination therapy, bevacizumab was administered for 3 cycles of tri-weekly maintenance therapy but was discontinued because of general fatigue. She has been alive without evidence of recurrence for 20 months since her initial surgery.

### Pathological examination

**Fig. 3:**
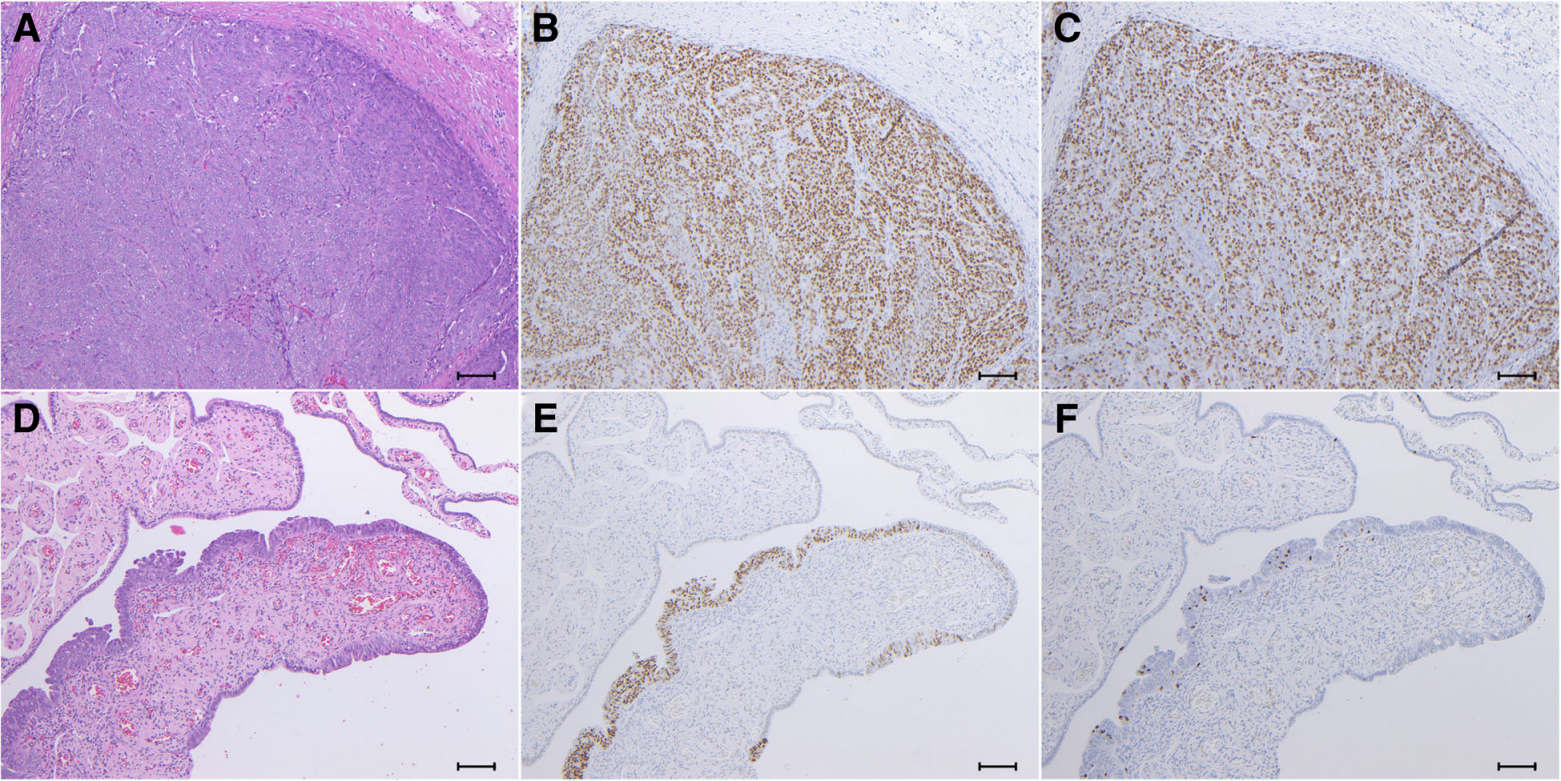
Histological images of **a**, **b**, **c** the retroperitoneal HGSC and **d**, **e**, **f** STIC examined by **a**, **d** hematoxylin and eosin staining, immunohistochemical staining for **b**, **e** p53 and **c**, **f** Ki-67. All panels are shown at power field of × 100. Scale bars show 100 μm

Macroscopically, the retroperitoneal tumor measured 80 × 55 × 35 mm in size and was divided into solid and cystic parts. The rectum and the peritoneum separated the tumor from the peritoneal cavity (Fig. 2c). The cyst part covered with a thick wall included bloody serous fluid. Removed genital organs (e.g. uterus, fallopian tubes, and ovaries) presented no abnormal gross findings except for uterine fibroids. Microscopically, a cyst wall which was composed of fibrous tissue contained hemosiderin-laden macrophages. A solid part of the tumor, characterized by extensive atypical nuclei and lace-like pattern by coalescence of papillae, revealed high-grade serous carcinoma (Fig. 3a). Although the tumor was invasive into rectal mascularis propria and adjacent fat tissues, the surgical margin, peritoneal invasion, and lymphovascular involvement were negative. In addition, neither cancer metastasis nor endosalpingiosis were identified in the lymph nodes in the adjacent fat tissue. There were no invasive lesions in genital organs but STIC lesion was detected at the right fallopian tube (Fig. 3d).

The cells of the retroperitoneal tumor and STIC were immunohistochemically positive for p53 (Fig. 3b & e). Ki-67 was diffusely and partially positive in the tumor and STIC lesions, respectively (Fig. 3c and f).

### Target-gene sequencing of microdissected cancer cells from the retroperitoneal tumor

The retroperitoneal tumor sample was immediately separated from surgical specimen, embedded in Tissue-Tek O.C.T. compound (Sakura Finetek) in a Tissue-Tek Cryomold (Sakura Finetek), and frozen in liquid nitrogen. Serial 8-μm-thick frozen sections were mounted on MMI Membrane Slides (Molecular Machines & Industries), fixed with 100% methanol, and stained with toluidine blue. We performed laser-microdissection (LMD) using the MMI CellCut system (Molecular Machines & Industries) to isolate tumor cells [14], followed by DNA extraction.

Somatic mutations were investigated by target gene sequencing in the retroperitoneal tumor. We selected 120 genes that were frequently mutated in ovarian and endometrial cancers from the TumorPortal website [14, 15] and involved in the homologous recombination repair pathway. We performed target sequencing of the 120 genes with a pool and capture method described in our previous study [14, 16]. The DNA libraries from the retroperitoneal tumor and the peripheral blood samples were sequenced via Illumina HiSeq 2500 platform in a rapid run mode with a 2 × 100 bp paired-end module. Referring to sequencing data from peripheral blood as a control, we called somatic mutations using Strelka software [17].

Somatic mutations detected in retroperitoneal tumor were listed in Table 1. Among six non-silent somatic mutations, a mutation of *TP53* (c.536A > G, p.179H > R) showed the highest mutant allele frequency (MAF) of 0.94. A nonsense mutation of *BRCA2* (c.6385G > T, p.2129E > X) also showed high MAF of 0.84. The MAFs of these two mutations were significantly higher than 0.5 (binomial test, *P <* 0.0001), suggesting the presence of either loss-of-heterozygosity events or homozygous mutations at these sites. We detected no obviously pathogenic mutations in the patient’s germline data obtained by sequencing for her blood cells.

**Table 1:** Mutations detected by target-gene sequencing of the retroperitoneal high-grade serous carcinoma

Gene	Amino acid (DNA) substitution	No. wild-type reads	No. mutant reads	MAF
*TP53*	179H > R (536A > G)	3	50	0.94
*BRCA2*	2129E > X (6385G > T)	17	89	0.84
*MLL4*	2009A > V (6026C > T)	21	39	0.65
*SIGLEC9*	Y117H (349T > C)	33	33	0.50
*MUC6*	1758H > L (5273A > T)	179	15	0.08

*MAF* denotes mutant allele frequency

### Deep sequencing of variant sites

To explore the genetic relevance between the retroperitoneal tumor and STIC lesion, deep sequencing was performed focusing on the two variant sites (*TP53*: c.536A and *BRCA2*: c.6385G) which were the most dominant in the retroperitoneal tumor. To obtain the STIC lesion, LMD was also performed using formalin-fixed paraffin-embedded (FFPE) tissue sections of 10-μm thickness. For LMD experiment, one per ten serial slides (*n* = 1, *n* = 11, etc.) was immunostained for P53. DNA samples derived from the retroperitoneal tumor and STIC were amplified by PCR reactions using the following oligonucleotide primers. Forward and reverse primers for *TP53* c.536A > G [p.179H > R] were 5’-CTGCTCACCATCGCTATCTG-3′ and 5’-CACATGACGGAGGTTGTGAG-3′, respectively. For *BRCA2* c.6385G > T [p.2129E > X], forward and reverse primers were 5′- CTGCTCACCATCGCTATCTG-3′ and 5’-CACATGACGGAGGTTGTGAG-3′, respectively. The PCR products were subjected to a deep sequencing via Illumina MiSeq v2 platform with a 2 × 150 bp paired-end module. The tag counts of paired-end sequences supporting the reference and mutant alleles were measured by using only high confidence base calls (base quality > 20) at the mutation sites.

As a result, the somatic mutations of *TP53* and *BRCA2* were shared between the retroperitoneal tumor and STIC lesion (Table 2). The MAFs of these two mutations were lower in STIC lesion. There is a possibility that STIC lesion was inadequately isolated from surrounding normal tubal epithelium, stroma and infiltrating immune cells through LMD, because the area of STIC lesion was limited.

**Table 2:** Deep sequencing information of the tumor and STIC

Targeted gene	Amino acid (DNA) substitution	Sample	No. wild-type reads	No. mutant reads	MAF
*TP53*	179H > R (536A > G)	Tumor	1685	7383	0.814
		STIC	152,901	110,704	0.420
*BRCA2*	2129E > X (6385G > T)	Tumor	6162	39,716	0.866
		STIC	96,243	86,018	0.472

*MAF* denotes mutant allele frequency

## Discussion

In this report, we presented HGSC existing entirely in retroperitoneal space without intraperitoneal malignant lesion except STIC in the fallopian tube, and demonstrated the genetic relationship between retroperitoneal HGSC and STIC lesion. In addition, extremely high MAF of the two mutations suggested that mutations might occur at the early stage of tumorigenesis, and it seemed reasonable to interpret that the origin of the retroperitoneal HGSC might be STIC.

To date, seven case reports of retroperitoneal HGSC are retrieved by searching NCBI PubMed service with the keywords of “retroperitoneal serous carcinoma” and “serous carcinoma & retroperitoneum” (Table 3) [7–13]. All patients were female, and onset age ranged from 11 to 75. In these reports, locations of the tumors varied from pelvis to upper abdomen in the retroperitoneal space. To explain how HGSC arose in retroperitoneal space, some theories represented by metaplasia of coelomic mesothelium and supernumerary ovary were discussed [7–12]. Unfortunately, no detailed analysis was performed in these reports to identify the pathogenesis of retroperitoneal HGSC and the origin of retroperitoneal HGSC remains unclear. In the two reports, bilateral salpingooophorectomy (BSO) was performed but there was no description about coexisting STIC. As shown in the article focused on the pathogenesis of epithelial ovarian cancer [18], a small STIC is frequently missed. In this case, STIC was diagnosed by a skilled gynecological pathologist (TM) based on morphological appearance and immunostaining for p53. For more accurate diagnosis of STIC, p16 was shown to be a useful biomarker in identifying STIC, separate from morphologically normal fallopian tube epithelium or HGSC [19, 20]. Based on the results in earlier studies, we attempted immunostaining for p16. However, all sections having the STIC lesion were used for performing LMD, which eliminated the opportunity of immunostaining for p16 or any further examination.

**Table 3:** Previous case reports of retroperitoneal HGSC

Author (year)	Age	Sex	Tumor size (cm)	Location of the retroperitoneal tumor	Prognosis
Ulbright (1983) [7]	11	female	18 × 13 × 11	Adherent to posterior pubic symphysis, involving the right retroperitoneum	N/A
Caruncho (1993) [8]	49	female	9 × 6 × 5	From the left ureteropyelic junction to the upper limit of the previs	N/A
Kurosaki (1998) [9]	38	female	6	Adherent to the lower pole of the right kidney	24 M alive
Kaku (2004) [10]	44	female	6 × 3.5 × 3	Surrounded by the left kidney, the aorta and the psoas major muscle	23 M alive
Demir (2007) [11]	40	female	15 × 13 × 10	In the right suprarenal fossa	N/A
Iura (2009) [12]	66	female	20 × 9.5 × 8.5	Adjacent to ascending colon, from lower limit of the liver to the ileocecum	32 M alive
Arichi (2011) [13]	75	female	4.8 × 5 × 5	Attached to the right kidney and the liver	6 M alive
Present case	58	female	8 × 5.5 × 3.5	In the mesorectum	16 M alive

N/A denotes not available

There are some theories about how STIC cells were transferred into the retroperitoneal space without peritoneal spread. Firstly, STIC cells could have migrated by lymphovascular metastasis in spite of “intraepithelial carcinoma” as Schneider et al. have described. They claimed that STIC should be regarded as a malignant lesion with metastatic potential by presenting that STIC cases accompanied lymph node metastases without any other intra-abdominal/peritoneal spread [21]. In this case, there were no evidence of the lympovascular invasion pathologically in surgical specimens including fallopian tubes. The patient was incompletely staged for peritoneal disease (or ovarian cancer), lacking pelvic and para-aortic lymphadenectomy, omentectomy, and peritoneal biopsy. Some articles have demonstrated microscopic metastasis at components for FIGO staging in early stage ovarian cancer. In the study of occult metastasis, lymphatic and omental/peritoneal involvement of serous carcinoma was detected at the rate of 17% (12/69) and 4% (3/69), respectively [22]. Another study reported that microscopic metastasis was found at pelvic peritoneum or omentum in two (9%) of 23 patients with apparent early serous ovarian cancer [23]. However, in apparent early stage epithelial ovarian cancer, random peritoneal biopsy and omentectomy beyond careful inspection of peritoneum was considered to have little significance [24]. However, we could not further assess the metastasis of STIC cells.

Secondly, tubal epithelial cells could have implanted directly into the mesentery, which resulted in endosalpingiosis. Kurman et al. have described the association between proliferative tubal epithelium and low-grade serous tumor and proposed the possibility that endosalpingiosis results from implantation of tubal epithelial cells [25]. Besides, it was reported that endosalpingiosis could be the origin of serous carcinoma in the mesentrium. McCoubrey et al. pathologically demonstrated the transformation from benign ciliated serous-type epithelium to well-differentiated serous carcinoma in a cystic endosalpingiosis in the mesosigmoid [26]. Combining these studies, we reasoned that *TP53*-mutated tubal epithelial cells developed into HGSC in the mesocolon via endosalpingiosis. In addition, our inference could be theoretically supported by the concept of “precursor escape,” which was recently raised by Crum [27]. Based on this novel concept, *TP53*-mutated tubal epithelial cells already have the potential to be the precursor of HGSC at other than tubal epithelium since they were p53 signature or early serous proliferations. In this case, one possibility is that p53 signature might have migrated into the mesentery and resulted in HGSC, while p53 signature in the fallopian tube grew into STIC.

## Conclusions

We presented a case of retroperitoneal HGSC possessing genetic relationship with STIC, suggesting that STIC is the precursor of retroperitoneal HGSC. When the tumor lying in the retroperitoneal space was diagnosed with HGSC pathologically, complete resection of the tumor with BSO and elaborated pathological examination of fallopian tubes should be recommended.

## Abbreviations

BSO: Bilateral salpingo-oophorectomy
CA125: Cancer antigen 125
CT: Computed tomography
HGSC: High-grade serous carcinoma
MRI: Magnetic resonance imaging
PCR: Polymerase Chain Reaction
STIC: Serous tubal intraepithelial carcinoma
